# The relationship between religious coping with body image concern among patients on hemodialysis: the mediating role of self-care

**DOI:** 10.3389/fpsyg.2025.1498416

**Published:** 2025-10-31

**Authors:** Hamid Sharif-Nia, João Marôco, Erika Sivarajan Froelicher, Badri Jaafari, Mozhgan Moshtagh, Fatemeh Khoshnavay Fomani, Amir Hossein Goudarzian, Omolhoda Kaveh

**Affiliations:** ^1^Psychosomatic Research Center, Mazandaran University of Medical Sciences, Sari, Iran; ^2^Department of Nursing, Amol Faculty of Nursing and Midwifery, Mazandaran University of Medical Sciences, Sari, Iran; ^3^Intrepid Lab, ECEO, Universidade Lusófona & CETRAD, UTAD, Lisbon, Portugal; ^4^Department of Physiological Nursing, School of Nursing, University of California, San Francisco, San Francisco, CA, United States; ^5^Department of Epidemiology and Biostatistics, School of Medicine, University of California, San Francisco, San Francisco, CA, United States; ^6^Student Research Committee, Shiraz University of Medical Sciences, Shiraz, Iran; ^7^Social Determinants of Health Research Center, Birjand University of Medical Sciences, Birjand, Iran; ^8^Nursing and Midwifery Care Research Center, School of Nursing and Midwifery, Tehran University of Medical Sciences, Tehran, Iran; ^9^Student Research Committee, Mazandaran University of Medical Sciences, Sari, Iran; ^10^School of Nursing and Midwifery, Tehran University of Medical Sciences, Tehran, Iran; ^11^Department of Nursing, Sari Faculty of Nursing and Midwifery, Mazandaran University of Medical Sciences, Sari, Iran

**Keywords:** body image, renal dialysis, religious coping, hemodialysis, Iran

## Abstract

**Objective:**

Exploring the factors that contribute to body image concerns among patients on hemodialysis is imperative. This cross-sectional study investigates whether self-care mediates the relationship between religious coping and body image concerns.

**Methods:**

A total of 398 patients completed the Littleton’s Body Image Concern Inventory Questionnaire, Assessment of Self-care Behaviors with Arteriovenous Fistula, and Religious Coping Questionnaire between February and May 2023 at a major comprehensive hemodialysis center in Iran.

**Results:**

The mean age of patients on hemodialysis was 56.97 (SD = 13.48). The model explained 23.5% of the variation observed in body image concern (*R^2^* = 0.235, *p* < 0.001). However, the mediation effect of self-care on body image was not statistically significant (*β* = 0.13, *p* = 0.434). In contrast, a mid-sized significant direct effect of religious coping on body image concerns was observed (*β* = 0.13, *p* < 0.001).

**Conclusion:**

The study contributes to the existing literature on the relationship between religious coping, body image concern, and self-care in various populations, such as overweight and obese individuals, high school students, and young females. The findings highlight that religious coping has directl relationship with body image concerns among patients on hemodialysis, while the mediating role of self-care was not supported. These results underscore the need for further research and targeted interventions that consider spiritual coping to improve body image outcomes in this population.

## Introduction

According to some estimates, the increase in the older population and the incidence of diabetes, hypertension, and obesity could raise the prevalence of End-Stage Renal Disease (ESRD), and its treatment burden by 2030 worldwide (Seyedzadeh et al., 2022; Thurlow et al., 2021). These patients must adhere to treatments such as hemodialysis, surgeries, and transplants to survive. Experiencing such conditions can disturb their functions, mental health, and body image (Celik et al., 2022; Sharif Nia, et al., 2022a). Body image is a subjective concept and is the aggregate of multiple dimensions, including feelings, cognitive evaluations, and bodily behaviors influenced by social norms that affect individual identity (Rodgers et al., 2023).

According to sociocultural theories, individuals internalize appearance norms and ideals during their life. Therefore any deviation from those ideals based on their self-concept can be associated with dissatisfaction or body image disturbance (Vartanian and Hayward, 2018). In line with these theories, changing functions or losing organs can affect body image leading to a threat to identity and an increased incidence of mental disorders. Accordingly, patients with ESRD are susceptible to experiencing body image disturbance due to special clinical conditions, changes in lifestyle and social roles, and self-efficacy (Sharif Nia, et al., 2022a). Considering that body image is associated with significant impacts on the results of treatments in chronic diseases, identifying its predictors would be worthwhile in designing intervention strategies (Lamarche et al., 2020).

Several studies have shown that body image concerns are highly prevalent among patients on hemodialysis, impacting their psychological well-being and quality of life (Marki et al., 2023; Yang, 2024). These patients often experience distress due to changes in physical appearance related to arteriovenous fistulas, skin changes, and fluctuations in body weight, which can exacerbate feelings of social isolation and depression (Marki et al., 2023). Gender differences have also been noted, with women often reporting higher body image dissatisfaction than men in this population (Marki et al., 2023). In the context of chronic illness, these concerns can further interfere with treatment adherence and self-care behaviors, underscoring the need to understand body image within the specific context of hemodialysis (Daniel et al., 2021).

### Effects of religious coping on body image

The effectiveness of religiosity on health and well-being has been revealed due to its positive roles in managing chronic conditions (Permana, 2018). Religious beliefs may emphasize body appreciation, which predicts engaging in health-promoting behaviors and taking care of one’s body, motivating people to pay attention to body functionality, which protects them from concerns about negative thoughts and appearances (Guest et al., 2019; Sharif Nia et al., 2017). Religious coping facilitates adaption to adversities, helping patients with chronic diseases accept themselves and find solutions to manage stressful situations and reduce psychological effects (Permana, 2018; Sousa et al., 2017).

Religious coping provides individuals with coping resources that have positive relation with overall well-being. These resources may include a sense of meaning and purpose, social support within religious communities, and a framework for understanding and interpreting life events (Surzykiewicz et al., 2022). By using religious coping strategies effectively, individuals may be more hopeful and likely to accept their illness, which may lead to taking an active role in their treatment and managing the consequences of the disease by addressing body image concerns and improving self-care behaviors (Celik et al., 2022; Surzykiewicz et al., 2022).

However, achieving adaptation depends on the individual’s attitudes towards God (positive/negative) and their perceptions (supportive/non- supportive) regarding relationships with the Lord (Dolcos et al., 2021; Permana, 2018). Based on the evidence, religious coping has two aspects. Therefore, it could have a dual function (adaptive/non-adaptive) to cope with the conditions influencing self-care behaviors (doing or avoiding) and positive or negative body image (Dolcos et al., 2021).

### Mediating role of self-care

Self-care is the ability to engage in behaviors necessary to modify adverse conditions and mitigate the consequences of chronic disease in order to preserve or promote health and quality of life (Santana et al., 2020; Tok Yildiz and Kaşikçi, 2020). Engaging in self-care behaviors is a way for individuals to actively manage their physical, emotional, and psychological well-being (Rogon et al., 2017; Sharif Nia et al., 2022a). These behaviors help patients to be independent and empowered to manage adverse conditions and social pressures that may lead to a positive body image (Santana et al., 2020).

Such individuals can experience improved body satisfaction, increased self-esteem, and reduced levels of body image concern. Self-care practices can also promote a sense of self-acceptance and self-compassion that are likely to positively affect body image perceptions (Rogon et al., 2017; Sharif Nia et al., 2022a). Previous research has indicated a significant relationship between religious coping and self-care behaviors (Sharif Nia et al., 2017). Therefore, we can consider the hypothesis that self-care mediates the relationship between religious coping and body image concerns.

### Theoretical framework

Theoretical support for the present study arises from Pargament’s religious coping framework (Surzykiewicz et al., 2022). Based on his definition, finding ways for coping is searching for significance and meaning during stressful conditions (Surzykiewicz et al., 2022). Religion facilitates conserving the most important and critical things in human life (Xu, 2015). According to Pargament’s framework, religious coping is a dynamic process that impacts health through multiple functions (Abu-Raiya, 2022). Religious beliefs help individuals manage their emotional responses to illness, actively engage in treatment, find support, and find meaning and resourcefulness (Cummings and Pargament, 2010; Xu, 2015).

### Purpose

This study was done with the aim of relationship among religious coping, body image concern, and self-care among patients on hemodialysis. Following hypothesis were considered:

*Q1*. There is a relationship between religious coping (positive and negative) and body image concerns among patients on hemodialysis.
*Q2*. There is a relationship between religious coping (positive and negative) and self-care among patients on hemodialysis.
*Q3*. Self-care mediates the relationship between religious coping (positive and negative) and body image concerns among patients on hemodialysis.

## Methods

### Design and participants

This study uses a cross-sectional study design with a convenience sampling method. The work has been reported in line with the STROCSS criteria (Mathew and Agha, 2021). The sample size of 266 was determined to be sufficient based on the sample size formula for structural equation modeling (Westland, 2010) and considering the anticipated effect size of 0.2, desired statistical power level of 0.8, number of latent variables 3, number of observed variables 49 and probability level of 0.05. In total, considering the possibility of participants who have incompletely filled the scales from a major comprehensive hemodialysis center in Sari, Iran.

### Sampling

A total of 280 patients completed the scales between February and May 2023. The study population consisted of patients with a diagnosis of ESRD undergoing hemodialysis. A convenience sampling method was used. Inclusion criteria were the ability to read and write in Farsi, and age 18 years and older. Exclusion criteria included alcoholism, mental, emotional and verbal problems based on the patient’s self-report, decreased level of consciousness, gastrointestinal diseases such as peptic ulcer and gastroesophageal reflux disease, and congestive heart failure.

During 3 months of data gathering, 360 patients were admitted to this medical center and 80 patients were excluded (mental disorders [32 patients], alcoholism [12 patients], gastroesophageal reflux [21 patients], and congestive heart failure [15 patients]).

### Instruments

Data collection tools included a demographic registration form, Littleton’s Body Image Concern Inventory Questionnaire (BICI), Assessment of Self-care Behaviors with Arteriovenous Fistula (ASBHD-AVF), and Religious Coping Questionnaire (R-COPE). The scales and questionnaires used in this study had a reliability of more than 0.7, indicating sufficient reliability for use (Table 1).

**Table 1:** Goodness of fit indices and reliability measures for the latent variables used in the mediation model.

Construct	Factor’s loadings range	Fit indices	Reliability measures
Selfcare	0.83 to 0.83	CFI = 0.996	*ω*_L1_ = 0.79
Selfcare prevention	0.75 to 0.99	TLI = 0.982	*α* = 0.91; *ω* = 0.93
Selfcare management	0.58 to 0.93	SRMR = 0.168 RMSEA = 0.13	α = 0.87; ω = 0.90
Body image	0.84 to 0.97	CFI = 0.985	ω_L1_ = 0.94
AppConc	0.88 to 0.93	TLI = 0.981	α = 0.90; ω = 0.92
HidFI	0.87 to 0.94	SRMR = 0.045	α = 0.86; ω = 0.89
NegBel	0.64 to 0.88	RMSEA = 0.65	α = 0.78 ω = 0.80
ApprOth	0.66 to 0.86		α = 0.73; ω = 0.75
Religious coping	−0.48 to 0.71	CFI = 0.955	ω_L1_ = 0.37
Positive coping	0.76 to 0.96	TLI = 0.947	α = 0.93; ω = 0.92
Negative coping	0.77 to 0.95	SRMR = 0.17 RMSEA = 0.13	α = 0.88; ω = 0.92

Self-Care, body image and religious coping are second order constructs. AppConc, Appearance concern; HidFI, Hiding the flaws; NegBel, Negative believe; ApprOth, Approval from others.

#### BICI

The 19-item BICI was first designed and validated by Littleton et al. (2005). This questionnaire examines a patient’s dissatisfaction, fear and embarrassment regarding appearance, checking and hiding perceived imperfections, and the interference of a patient ‘s fear of appearance with social performance. The response options were a five-point Likert scale ranging from never = 1 to always = 5 (Littleton et al., 2005).

The total score of the questionnaire can vary between 19 and 95, and the highest score indicates more dissatisfaction with the body image or appearance. The BICI has been translated into Persian and validated with satisfactory psychometric properties in an Iranian population (Aramesh et al., 2020). The reliability of BICI was calculated as *α* = 0.94 in a sample of Iranian patients needing hemodialysis (Sharif Nia et al., 2022a). The reliability of the BICI was calculated as *α* = 0.943 in the present study.

#### ASBHD-AVF

Hemodialysis patients’ self-care behaviors are evaluated by the ASBHD-AVF. This scale, which was created in Portuguese, has 16 items divided into two subscales: Subscale 1 (self-care in managing signs and symptoms, 6 items), and Subscale 2 (self-care in preventing complications, 10 items).

Responses to each question are based on a Likert scale with a maximum score of 5 (always practice self-care) and a minimum score of 1 (never practice self-care). Higher scores demonstrate that patients use the ASBHD-AVF for self-care more frequently. Internal consistency for the Portuguese scale and subscales 1 and 2 as determined by Cronbach’s alpha was 0.79, 0.79, and 0.72, respectively (Sousa et al., 2015). The reliability of ASBHD-AVF was calculated as α = 0.87 in a sample of Iranian patients on hemodialysis (Sharif Nia et al., 2022a). The validity and reliability of ASBHD-AVF were evaluated in a hemodialysis patient population and demonstrated acceptable psychometric properties (Sharif-Nia et al., 2024a,b). The reliability of the ASBHD-AVF was calculated as *α* = 0.872 in the present study.

#### R-COPE

Religious coping methods were investigated using R-COPE. This standard questionnaire had 14 items to measure positive and negative religious coping, and it was developed by Kenneth Pargament. Each positive and negative scale included seven options for religious coping test. The scoring method utilizes a Likert scale, from “not at all” to “many times.” Positive religious coping is a style of dealing with negative life events in which a person uses the evaluation and positive changes associated with God to deal with those events. A person believes that God will not abandon them, when confronting sad events. On the other hand, negative coping involves establishing an avoidant and insecure relationship with God. For example, one may believe that God will leave them alone in difficult moments. The reliability of R-COPE was established in an Iranian population (Goudarzian et al., 2019; Nesami et al., 2015). The reliability of the R-COPE was calculated as α = 0.707 in the present study. The scale items are shown in Appendix 1.

### Ethical considerations

The Ethics Committee of Mazandaran University of Medical Sciences approved this study (ethics code: IR. MAZUMS. REC.1402.477). The patients were informed that participation in the study was voluntary and had the objectives and procedures fully explained to them. Every participant was also provided with written, fully informed consent. All participants received assurances regarding the privacy of the data and the findings that would be shared and published.

### Data analysis

Evidence of validity related to the internal structure of the constructs was obtained by means of confirmatory factor analysis (CFA) on the polychronic correlation matrix using either the DWLS estimator (for CFA) or robust ML (for the mediation model) present in lavaan package for the R statistical system (2021). The study employed various goodness-of-fit indices, namely the Chi-square statistic (χ^2^), comparative fit index (CFI), Tucker-Lewis index (TLI), root mean square error of approximation (RMSEA), and standardized root mean square residual (SRMR), to assess the fit of the model.

According to the established criteria (Hu and Bentler, 1999; Marôco, 2021), an acceptable model fit was indicated by CFI and TLI values greater than 0.90, and RMSEA and SRMR values less than 0.06 and 0.08, respectively. To evaluate the reliability of the measures, internal consistency analyses were conducted using the Sem Tools R package (Hove et al., 2019). Specifically, Cronbach’s alpha coefficient (*α*) (Cronbach, 1951) and coefficient omega (*ω*) (McDonald, 2013) were computed for each factor.

Reliability of second order constructs was assessed with ω_L1_ (McDonald, 2013). A satisfactory level of internal consistency was indicated by alpha and omega values greater than or equal to 0.7, as recommended by Marôco (2014). The mediation model was fitted and tested using the lavaan package, and standard goodness-of-fit indices were used for model evaluation. Additionally, R^2^ values were calculated for both the mediator and criterion variables.

## Results

The mean age of cancer patients was 56.97 (SD = 13.48). Most of the patients were men (56.1%, *n* = 157) and married (90.4%, *n* = 253).

### Evidence of validity and reliability of constructs

Goodness of fit indices and reliability measures, as described in the methods section, the different constructs are presented in Table 1. Overall, all constructs displayed evidence of good factorial validity and reliability, ensuring proper validity and reliability of the data used in the mediation model.

**Figure 1:**
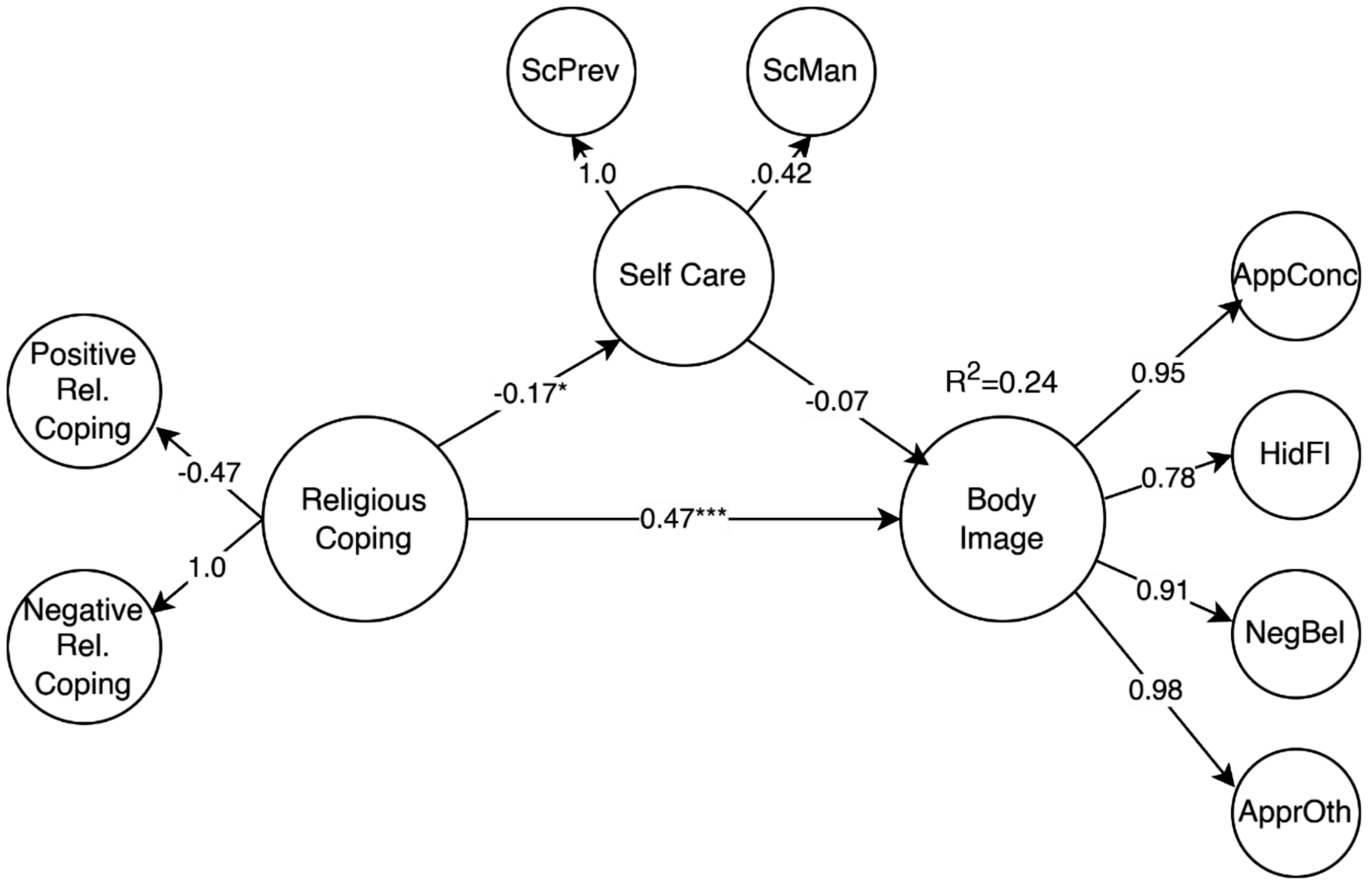
Religious coping direct effect on body image concerns and mediation through self-care. Values are the standardized loadings from second order constructs to first order constructs (all statistically significant) and structural regression weights (*p < 0.05, **p < 0.01, ***p < 0.001). AppConc, Appearance concern; HidFI, Hiding the flaws; NegBel, Negative believe; ApprOth, Approval from others; ScPrev, Selfcare prevention; ScMan, Selfcare management.

The mediation model’s results are presented in Figure 1, showing limited goodness of fit (CFI = 0.862, TLI = 0.852, SRMR = 0.08, RMSEA = 0.08), indicating that the current model may not fully capture the complexity of relationships among religious coping, self-care, and body image concerns. The model explained 23.5% of the variation observed in body image concern (*R^2^* = 0.235, *p* < 0.001). However, the mediation effect of self-care on body image concerns was not statistically significant (*β* = 0.13, *p* = 0.434). In contrast, a mid-size significant direct effect of religious coping on body image concerns was observed (*β* = 0.13, *p* < 0.001).

**Figure 2:**
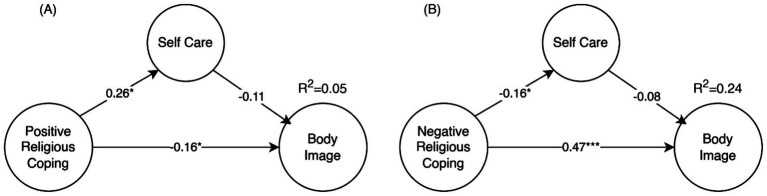
Positive religious coping direct effect on body image concerns and mediation through self-care **(A)** and Negative religious coping direct effect on body image concerns and mediation through self-care **(B)**. Values shown are the standardize structural regression weights (*p < 0.05, **p < 0.01, ***p < 0.001). For clarity factor loadings and first order factors are omitted.

Individual models were fitted for Positive and Negative Religious Coping due to the poor overall reliability of religious coping (ω_L1_ = 0.37) and the imbalance of positive vs. negative coping manifestations. Figure 2 summarizes the results of both models. The Positive Religious Coping model showed poor fit (CFI = 0.886, TLI = 0.877, SRMR = 0.066, RMSEA = 0.076) and only explained 4.8% of the variation in body image concerns. Although Positive Religious Coping had a significant effect on self-care (*β* = 0.26, *p* = 0.023), the mediation effect was not significant (*β* = −0.03, *p* = 0.25). Conversely, the direct effect from Positive Religious Coping to body image concerns was significant (*β* = −0.158, *p* = 0.01), although with a small effect size.

The model for Negative Religious Coping still had a poor fit (CFI = 0.855, TLI = 0.844, SRMR = 0.079, RMSEA = 0.077), but it explained 24.2% of the body image concerns. The mediation effect was not significant (*β* = 0.012, *p* = 0.419). The effect of negative religious coping on self-care was small and negative (*β* = −0.16, *p* = 0.024), and the most significant effect was the positive direct effect of negative religious coping on body image concern (*β* = 0.47, *p* < 0.001).

**Figure 3:**
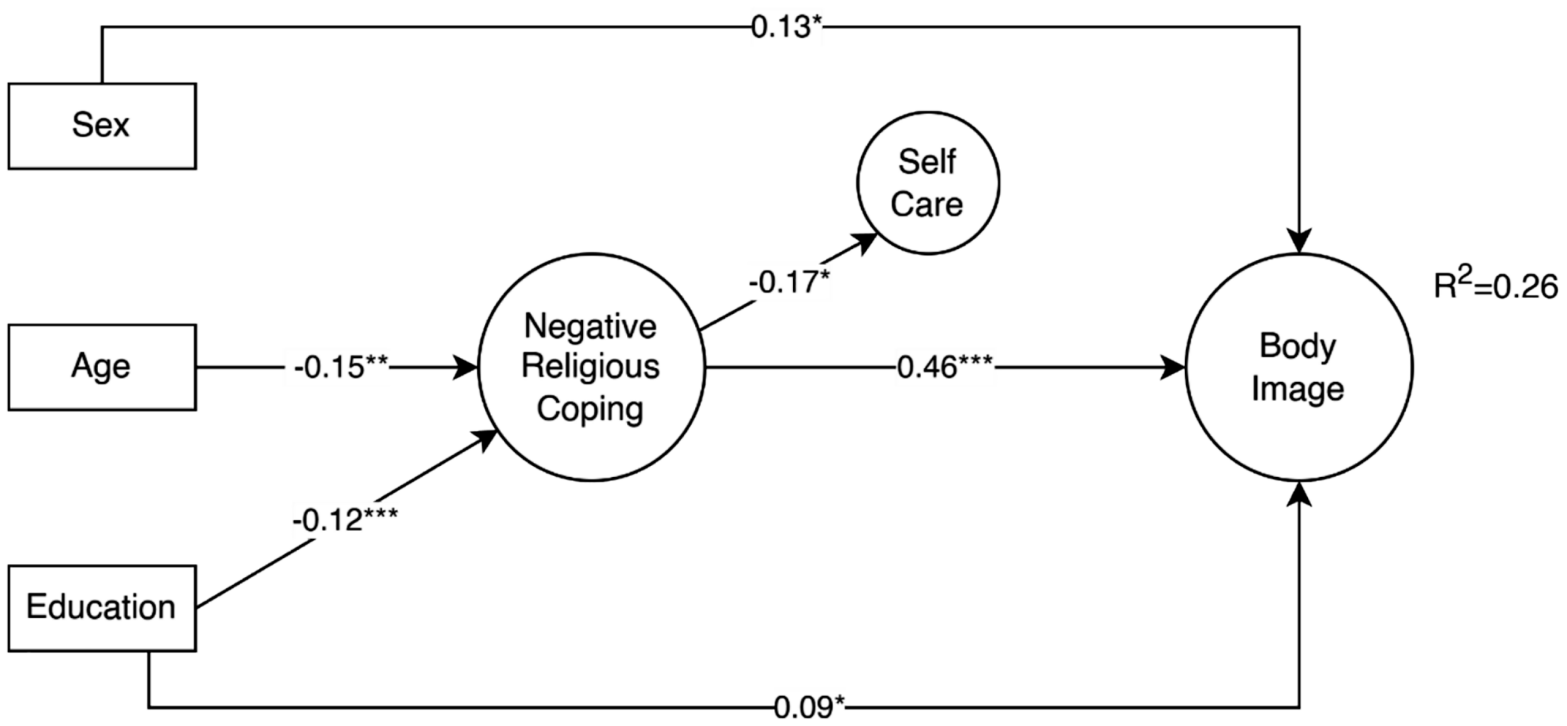
Effects of sex, age and education on the negative religious coping effects on body image concerns and mediation through self-care. For simplicity only statistically significant paths (*p* ≤ 0.05) are shown.

The effects of socio-demographic variables like gender, age and education are shown in Figure 3. Both age (*β* = −0.15, *p* = 0.01) and education (*β* = −0.12, *p* < 0.001) had a negative and significant effect on negative religious coping. Both sexes (0-men, 1-women, *β* = 0.13, *p* = 0.034) and education (*β* = 0.09, *p* = 0.05) had positive and significant effects on body image concern. The effect of education was also not mediated by negative religious coping (*β* = −0.06 ns). However, these direct as well as indirect effects mediated through negative religious coping; only explained 2% more of the variation in body image concerns (*R^2^* = 0.26) compared with model B from Figure 2 (*R^2^* = 0.24).

## Discussion

In the present study, we aimed to investigate the mediating role of self-care and the relationship between religious coping and body image concerns among patients on hemodialysis. The current study revealed that age and education had a negative and significant effect on negative religious coping. This means that the negative style of religious coping decreased in those patients who were older or had higher educational levels. Many studies have indicated that the religious coping style is dependent on individual age, gender, educational level, and other demographic characteristics (Kulakçı-Altıntaş and Ayaz-Alkaya, 2020; Ooi et al., 2023).

It is believed that younger people may participate less in religious activities (e.g., daily prayers) compared to older individuals, and religion is more important for the elders (Stearns et al., 2018). It has also been demonstrated that a higher level of education leads to a lower level of religiosity (Rachmatullah et al., 2019). However, people from different geographical and cultural settings exhibit variations in considering themselves to be religious persons (Rachmatullah et al., 2019). This may influence the religious coping strategy they choose.

### Religious coping and self-care

Based on the main findings of the current study, religious coping had a significant effect on self-care in patients on hemodialysis. The correlation between religious coping and self-care has been identified in different study samples (e.g., medical students Sharif Nia et al., 2017), patients with cancer (Goudarzian et al., 2019), patients who have diabetes mellitus (Dewi et al., 2022), and patients needing hemodialysis (Sharif Nia et al., 2022a,b). However, the current study proposed the religious coping and self-care relationship by implementing a predictive approach in a sample of patients on hemodialysis.

Hemodialysis is a life-saving medical procedure for individuals with end-stage renal disease, and self-care is essential to manage their condition and improve their quality of life. Religious coping refers to the ways in which individuals use their faith and religious beliefs to cope with the challenges and stressors they face (Hamid Sharif Nia, Daniyal Kohestani, et al., 2022a). Religious coping can create a positive attitude in patients with chronic health problems and therefore motivate and empower them to promote self-care behaviors (Rezaeipandari et al., 2023). Furthermore, religion can provide emotional support and comfort to patients needing hemodialysis.

Engaging in religious practices, such as prayer or meditation, may help reduce anxiety, depression, and stress, which can positively impact motivation to engage in self-care behaviors (Chatrung et al., 2015). Religious beliefs often emphasize the importance of resilience and tolerance in the face of adversity. Patients needing hemodialysis who draw strength from their faith may be more resilient and better able to adhere to their self-care routines including dietary restrictions, medication management, and regularly attending dialysis sessions (Sharif Nia et al., 2017). Positive religious coping predicts mental and social health, so patients on hemodialysis who cope positively and are active members of a religious community may have access to a better support system, such as help with transportation to dialysis centers or grocery shopping, that can facilitate their self-care.

In addition, religious coping may help patients manage pain and discomfort by providing a sense of transcendence and detachment from their physical suffering (Dewi et al., 2022). Religion can provide a sense of meaning and purpose in life. Patients needing hemodialysis who find meaning in their lives based on their religious beliefs may be more motivated to take care of themselves and adhere to their treatment plans, as they see their struggles as part of a larger spiritual journey. Belief in a higher power or divine intervention can provide patients on hemodialysis with a sense of control over their condition. This belief may encourage them to take an active role in their self-care, as they believe they are co-partners in their health journey with a higher power (Dewi et al., 2022; Sharif Nia et al., 2022b).

### Religious coping and body image concerns

A relationship between negative religious coping and body image concerns was identified in the current study. Studies indicate that negative religious coping and mistrust in God correlate with more stress and less positive impact (O'Brien et al., 2019; Pirutinsky et al., 2020). In this regard, the findings of a systematic review indicated that negative religious coping coupled with superficial faith in God was associated with a higher level of body image concerns (Akrawi et al., 2015). Religious coping can promote a positive body image by emphasizing inner qualities and the intrinsic worth of individuals.

Patients on hemodialysis who draw strength from their faith may view their bodies as temples or vessels for their souls, valuing their spiritual well-being above physical appearance. This perspective can boost self-esteem and reduce body image concerns (Yamani Ardakani et al., 2020). Some religious beliefs stress the acceptance of life’s challenges as part of a larger divine plan. Patients on hemodialysis who hold these beliefs may be more inclined to accept the physical changes associated with their treatment, such as vascular access scars or weight fluctuations. They may interpret these changes as tests or trials that they are meant to endure, reducing distress related to body image (Tiggemann and Hage, 2019).

Patients on hemodialysis can turn to religious practice and positive religious coping to deal with negative body image concerns and emotional distress related to their changes in physical appearance. Such coping strategies can improve mental well-being (Poulter and Treharne, 2021). Certain religious teachings emphasize the importance of inner beauty and character over appearance. Patients on hemodialysis who internalize these teachings may focus less on physical appearance and more on cultivating virtues and qualities that are valued in their faith, leading to greater contentment and reduced body image concerns (Wortmann, 2020).

Some religious traditions discourage excessive vanity and materialism, promoting humility and modesty. Patients on hemodialysis who adhere to these teachings may be less preoccupied with superficial beauty ideals and, consequently, experience fewer body image concerns related to societal standards. Religious coping can enhance psychological resilience, helping patients cope with the challenges of body image changes more effectively. By grounding their self-worth in their faith and finding strength in their beliefs, individuals may be better equipped to navigate the emotional and psychological impact of their physical changes (Sharif Nia et al., 2022b; Yamani Ardakani et al., 2020).

### Mediation role of self-care

In the present study, the mediating role of self-care in the relationship between religious coping and body image concerns was not statistically significant. Inconsistent with this finding, some studies have found a significant positive relationship between these two variables (Cook-Cottone, 2015; Sharif Nia et al., 2022a; Sharif Nia et al., 2022b). To the best of our knowledge, the findings of this study show that self-care as a mediating factor had no significant effects on body image concerns.

Differences in study design, methodology, and statistical analyses may have contributed to these disparate results. The inclusion of further variables or the use of different statistical techniques can influence the outcomes. For instance, confounding variables may compete with other variables to explain the study outcomes. Additionally, studies may have different participant demographics. Factors such as age, gender, cultural background, and the severity of health conditions can influence both self-care behaviors and body image concerns.

By the way, addressing the main issue of engaging in self-care behaviors, such as adhering to dietary restrictions, taking prescribed medications, attending hemodialysis sessions regularly, and managing comorbid conditions, can improve overall health and well-being. When patients feel that they are taking control of their health and effectively managing their condition, it can boost their self-esteem and reduce body image concerns (Sharif Nia et al., 2022a). Proper self-care can help manage physical symptoms associated with ESRD, such as fluid retention or electrolyte imbalances, which can impact physical appearance.

As self-care leads to better symptom management, patients may experience fewer visible changes in their bodies, reducing body image concerns (Lee et al., 2021). Self-care empowers hemodialysis patients to actively participate in their treatment and make decisions about their health. This sense of empowerment and control over their condition can mitigate feelings of helplessness and improve body image by emphasizing personal agency in managing health (Sharif Nia et al., 2022b).

While our findings revealed that positive religious coping did not significantly affect body image concerns, several potential explanations can be considered. First, in many cultural contexts, including Iran, positive religious coping may focus more on collective practices and spiritual endurance rather than on perceptions of physical appearance, limiting its direct relation with body image concerns (Sharif-Nia et al., 2024a,b). Second, body image issues in patients on hemodialysis may be more strongly influenced by visible treatment-related physical changes and symptom burden than by general positive religious beliefs or practices. Third, it is possible that measurement tools for positive religious coping and body image do not capture overlapping domains, reducing the likelihood of detecting a direct relationship (Ahmadi et al., 2018). Future qualitative studies may help uncover the nuanced ways in which positive religious coping may indirectly affect body image concerns through emotional resilience or acceptance in this population.

### Limitations

It’s important to note that the impact of religious coping on self-care and body image concerns can vary greatly from person to person. Some individuals may find great comfort and motivation in their religious beliefs, while others may not. This variability may affect the results of these studies. Many studies in this field use cross-sectional designs, which capture data at a single point in time. This design limits the ability to establish causality or identify changes in self-care behaviors and body image concerns over time.

Additionally, the limited goodness of fit observed in the current models highlights the need for further research to refine measurement models and explore additional variables that may contribute to understanding body image concerns in patients undergoing hemodialysis.

It is important to note that this study did not collect or include data on patients’ religious affiliation or level of religious belief, which could have significant relation with religious coping styles and body image concerns. The omission of these variables may limit the interpretation of the findings regarding the impact of religious coping. Future studies should include these demographic and belief-related variables to better understand the nuanced role of religious coping across different religious and spiritual backgrounds in shaping body image concerns among patients on hemodialysis.

Data collected through self-report questionnaires or interviews may be subject to response bias. Participants may underreport or overreport their self-care behaviors or body image concerns due to social desirability or memory recall issues. The study findings may primarily apply to the specific population under investigation and may not be applicable to other medical conditions or patient populations. Therefore, more studies with special attention to these limitations and larger samples should be conducted in the future. Nevertheless, the following conclusions can be drawn.

## Clinical implication

The findings of this study have important clinical implications for healthcare providers caring for patients on hemodialysis. Given that negative religious coping is directly associated with higher body image concerns, routine psychosocial screening should include assessments of patients’ religious coping styles to identify those at greater risk of distress. Incorporating spiritual care interventions, such as referrals to chaplaincy services or culturally sensitive counseling, may help patients reframe negative religious interpretations and reduce body image concerns. Additionally, healthcare providers can integrate discussions around body image concerns within routine care, emphasizing acceptance and coping strategies aligned with patients’ spiritual values to enhance overall well-being. Tailoring interventions that consider patients’ spiritual frameworks may improve engagement with self-care and adherence to treatment while addressing psychological distress related to body image.

## Conclusion

This study demonstrates that religious coping has a direct and significant impact on body image concerns among patients on hemodialysis, independent of self-care behaviors, with negative religious coping exerting a stronger relation than positive religious coping. Although the mediation role of self-care was not supported, the findings highlight that patients who engage in negative religious coping may experience heightened body image concerns, while positive religious coping may help alleviate these concerns.

These results underscore the complex interplay between spiritual coping mechanisms and psychological well-being in chronic illness. Addressing religious coping in clinical settings may provide an important pathway to support the mental health and body image of patients undergoing hemodialysis, beyond focusing solely on physical care and self-management strategies.

Future research should investigate interventions integrating spiritual care with psychological support to improve body image outcomes, considering individual variations in religious beliefs and practices. Additionally, longitudinal studies are recommended to clarify causal relationships and explore how changes in religious coping and self-care behaviors over time affect body image concerns in this patient population.

## Data Availability

The raw data supporting the conclusions of this article will be made available by the authors, without undue reservation.
