# Supplementary material for: The relationship between religious coping with body image concern among patients on hemodialysis: the mediating role of self-care
Source: Front Psychol. 2025 Oct 31;16:1498416. doi: 10.3389/fpsyg.2025.1498416 (PMC12615242; doi:10.3389/fpsyg.2025.1498416)
Supplement: Supplementary file 1 [file Table_1.docx]

**Appendix 1.** Religious coping scale

| 1. Looked for a stronger connection with God. |
| --- |
| 2.  Sought God’s love and care. |
| 3. Sought help from God in letting go of my anger. |
| 4. Tried to put my plans into action together with God. |
| 5. Tried to see how God might be trying to strengthen me in this situation. |
| 6. Asked forgiveness for my sins. |
| 7. Focused on religion to stop worrying about my problems |
| 8. Wondered whether God had abandoned me. |
| 9. Felt punished by God for my lack of devotion. |
| 10. Wondered what I did far God to punish me. |
| 11. Questioned God’s love for me. |
| 12. Wondered whether my church had abandoned me. |
| 13. Decided the devil made this happen. |
| 14. Questioned the power of God. |
